# When Will Reason and Judgment Prevail?

**Published:** 1900-11

**Authors:** 


### EDITORIALS.

### WHEN WILL REASON AND JUDGMENT PREVAIL?

It is with reluctance that we offer to our readers another editorial on tuberculosis. This subject has many times been presented and discussed in the pages of this Journal. It would, at first sight, seem that there remains nothing worth saying that has not already been said clearly and well. But still false views, erroneous opinions, and, indeed, sometimes, wilful and malignant misstatements on this subject are propounded and sent forth with the approval of some one holding the confidence or the attention of a portion of the public. Perhaps all of this is natural and should be expected, for it is impossible to educate the entire public to correct views on a question that has been misunderstood for generations. How difficult it has been, for example, to teach the public, and even some doctors, that tuberculosis is not usually acquired by inheritance.

Tuberculosis will be suppressed when the facts in regard to the disease are understood by the people. The way is clear. How do we know that it is clear? Because many breeders and dairymen have followed it, and have arrived with healthy herds at the goal they sought, leaving tuberculosis behind. It is necessary, however, that the FACTS disseminated in regard to tuberculosis shall in truth be the facts and not an aggregation of unfair and incomplete extracts from and prejudiced comments upon biased articles on this subject, compiled and distorted by a man who, though an expert on some questions associated with animal husbandry, is not a pathologist or bacteriologist, and who does not have special knowledge of the diseases of animals, but who is the possessor of a tubercular and run-down herd that he is anxious to sell. We are led to these reflections by a review of a short series of articles on tuberculosis, published some time ago by a weekly journal for the American stock-farmer, and by an editorial in the *Breeders' Gazette* (reprinted elsewhere in this issue) entitled "The Slaughter Cure for Tuberculosis." It is evident that the articles first referred to and the editorial were written by different men. The former were hazy with pseudo-science, studded with false premises and unsound deductions, and reflected the handiwork of the penny-a-liner or the poseur. The editorial is different. It most stupidly sets up a man of straw and then assails him viciously. But its author pursues tactics that are nasty and foul, even when fighting men of straw; he hits below the belt; he resorts to mayhem; he becomes hysterical, claws, scratches, and shrieks. His product reeks with gall and venom. He appears to feel that he is one chosen to prepare the whirlwind or to direct the shotgun quarantine that is to destroy all who "run amuck with tuberculin squirt-gun and pole-axe among the herds of farmers." Such a production at first excites mirth, and then sorrow—sorrow that such a generally reputable paper should accept and print on its first page, and back by its prestige, this accumulation of misinformation, insults, half truths, absolute errors, and balderdash. "Such a criticism is all very well, but can you refute the statements in the editorial?" some may say. Perhaps it is worth while, then, to analyze the article, not for the benefit of our well-informed readers, but for the good of the Sanderses, the Goodwins, etc., whom one occasionally meets.

1. Live-stock sanitary boards and veterinarians are not practising or recommending, and very few veterinarians ever did advise, the general destruction of all animals reacting to the tuberculin test. In many States a system similar to that originated by Bang is officially recommended, and owners of reacting cattle are encouraged to adopt it, as in Pennsylvania. In some States (Massachusetts, New Jersey, and others) only physically suspicious cattle are tested with tuberculin.

2. General herd tests are not compulsory in any State in America. In nearly all States provision is made for the relief of farmers who wish to eradicate tuberculosis from their herds, but established herds generally are not required to be tested.

Cattle of some classes are required to undergo and pass the tuberculin test when moving from one State to another. This is practically the only instance in which the test is compulsory.

In Illinois the board arranged "to conduct tests only where the owners of cattle made voluntary application for the same, and agreed to abide by the rules and regulations prescribed by the board for conducting such tests, save where, as a result of tests, the prevalence of disease was positively demonstrated to exist in other herds. *With one exception, all the tests made during the past year were conducted on the application of the owners.*" (Report of the Illinois Live-stock Commissioners, 1899, p. 23.)

There is absolutely no instance of general compulsory test at this time, nor is any one recommending such general tests. It was found in Massachusetts several years ago that this plan was not a desirable one. The unsatisfactory work done in Massachusetts was planned and conducted by a board made up in part of farmers. Hence, the slur about "unrestrained vets running amuck," etc., is entirely gratuitous and is empty clatter.

3. Bang and Nocard do not, it is true, recommend the general slaughter of reacting cattle. But in all fairness their recommendations and practices should be stated, since they are editorially commended as men who have most carefully studied the question and are moderate and sensible in their teachings. Both of these safe leaders advise in the strongest terms, and practice, the testing of infected herds with tuberculin and the separation of all reacting cattle. Those showing clinical signs of tuberculosis are killed, the other reacting animals are kept, practically in quarantine, as a separate herd. The milk from such cows is heated before it is permitted to be used.

Can farmers here practice this plan in ridding their herds of tuberculosis? If they cannot keep cattle profitably under these conditions it is useless to adopt or to advocate the Bang system.

4. As no one has ever claimed tuberculin to be infallible, the wonderful discovery announced by the *Gazette* that it is not infallible cannot count for much. One might as well say that "extensive experiments made on the Continent, in Britain, and in this country have shown that the speed of railroad trains is limited; they do not run 200 miles an hour, so why use them?" Use them because they afford the best means of transportation available. And tuberculin is used because it affords the best means of diagnosticating tuberculosis that is available. But, as the *Gazette* says, it must be placed in proper hands and used at the proper time. Surely, no one has ever said that it should be placed in improper hands and used at improper times! It is true that some advanced cases do not react to tuberculin. This is well known to all veterinarians, and guarded against, so that the advanced cases do not pass and are not permitted to do harm.

5. Tuberculosis is already scheduled as a contagious disease in the human family, and must be reported to the health officer along with smallpox, diphtheria, and the like in New York City and many other places in this country. Such reports are also required in a very large number of foreign cities. This regulation is strongly recommended by physicians, and is spreading. Moreover, the sanitaria for consumptives provide means for isolating many dangerous cases.

6. Tuberculosis is essentially, primarily, and absolutely a contagious disease, and is so recognized by all physicians. It spreads by direct or indirect contact.

7. If tuberculosis were such a disease as smallpox or scarlet fever it would do precisely what smallpox and scarlet fever do. These diseases have not exterminated the human race, nor have the plague, cholera, yellow fever, and diphtheria combined. Surely some of these diseases are contagious.

8. "Tuberculosis may be transmitted if the conditions are favorable." This is all that can be said of any contagious disease or any fatal influence, and is all that any one has ever claimed. A bullet will kill if the conditions are favorable—that is, if there is enough powder in the cartridge, if the gun is rightly aimed and fired at the right time, if the object is within range and has a vital part exposed, etc. In every outbreak of every disease, however virulent, there is a large proportion of escapes.

9. There is not a warm-blooded animal of any species that may not be killed by tuberculosis. No animal has complete immunity to this disease. It is all a question of degree. Some animals contract the disease readily because they furnish favorable conditions for the growth of the germ; other animals require prolonged exposure and large numbers of bacilli before they succumb.

To say that "the healthy system affords no habitat for the germs of tuberculosis" is to say that as soon as the germs of tuberculosis enter and remain, as they often do, the system becomes unhealthy. To use another simile, we may say that the healthy man never has pneumonia, for a man having contracted pneumonia is unquestionably ill. The essence of this point is that it is absolutely impossible to say that any person or bovine animal is proof against tuberculosis. Any of them may contract it if exposed. This is abundantly proven by the experience of every practising physician or veterinarian.

10. The air is not full of the germs of tuberculosis. It was formerly believed that the germs of tuberculosis were ubiquitous. This belief was but an empty view or opinion. It was never based on observation. No one has ever found tubercle bacilli widely distributed. For years scientists have been hunting for them, and have used the most accurate methods and perfect instruments that can be devised. What is the result? Are tubercle bacilli found in the air of our streets, in the carpets, cracks, and corners of the churches and theatres and on car windows? Not at all. Tubercle bacilli are found only about consumptives, human or bovine, places occupied by them and their products. That is all.

Is it asking too much of a paper to be accurate? If a paper screams its opinion at you with frantic gestures, is it too much to ask that these opinions be modern and not those discredited years ago? And if a paper persists in vomiting putrid editorials that are neither true nor up-to-date, should it not at least be polite enough to hide them among the advertising pages? Of course, we must admit, it is hard to be polite when it finds that a straw man set up with much labor and then kicked with force is filled with lead.

And now, finally, is it not fair to assume that the *Gazette* does not wish the ravages of tuberculosis to be checked, if it does not propose some plan for the accomplishment of this end and furnish some actual proof that the plan will work and has worked successfully? But from the above it must be clear that opinions from the *Gazette* on tuberculosis should hereafter be accompanied by the evidence in full. Perhaps it would be well to have the evidence sworn to.

